# Supplementary material for: The interactome of intact mitochondria by cross-linking mass spectrometry provides evidence for coexisting respiratory supercomplexes
Source: Mol Cell Proteomics. 2017 Dec 8;17(2):216–32. doi: 10.1074/mcp.RA117.000470 (PMC5795388; doi:10.1074/mcp.RA117.000470)
Supplement: Supplemental Data [file supp_RA117.000470_133922_0_supp_23978_fzffwf.zip › spectra_annotation/mito_spectra_annotation/94-1-2-1-12-1.pdf]

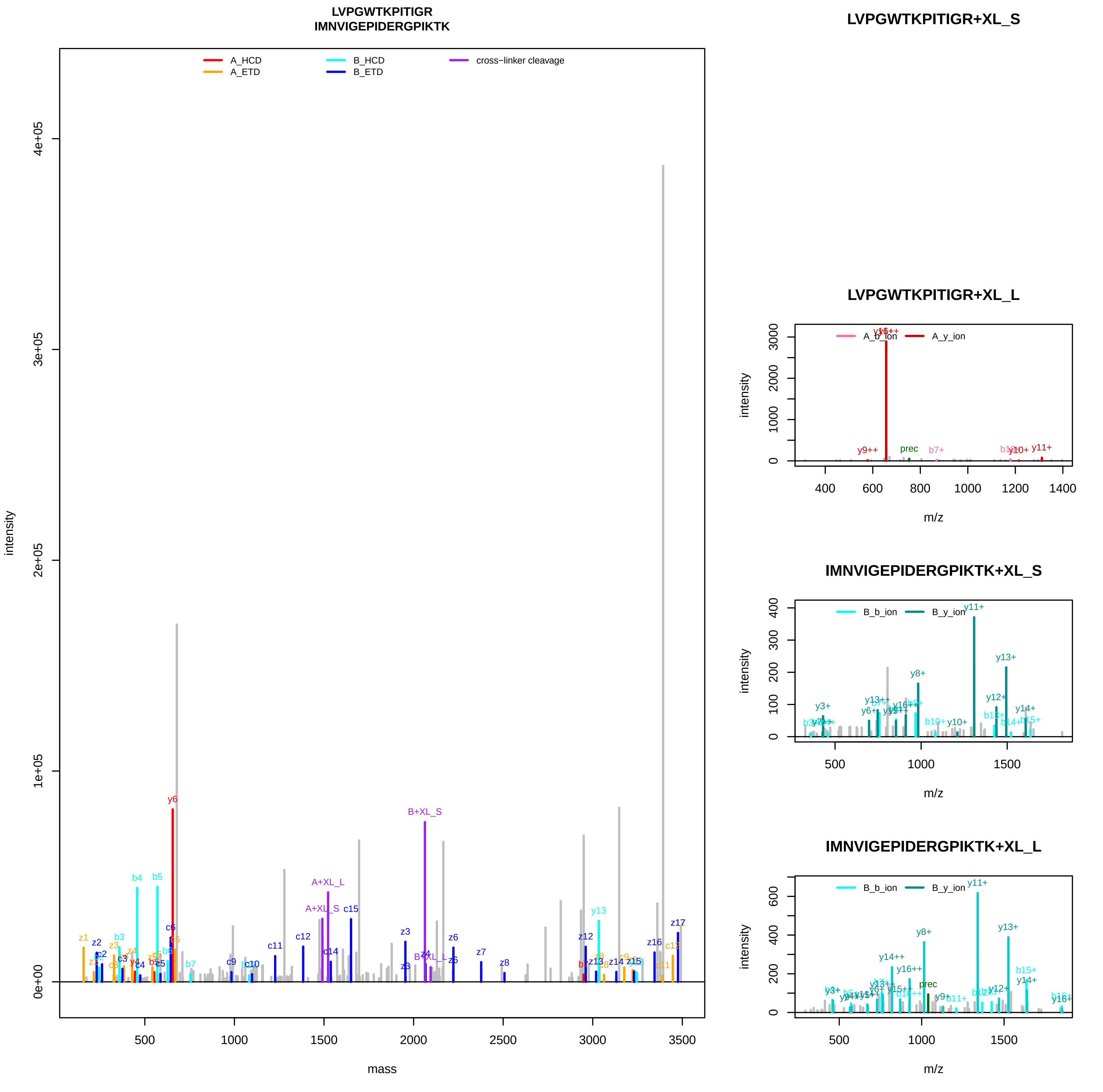
LVPGWTKPITIGR
IMNVIGEPIDERGPIKTK
A_HCD
A_ETD
B_HCD
B_ETD
cross−linker cleavage
intensity
mass
0e+00
1e+05
2e+05
3e+05
4e+05
500
1000
1500
2000
2500
3000
3500
y6
B+XL_S
A+XL_L
A+XL_S
b4
b5
c15
y13
z17
c12
c11
z3
z6
z7
z8
z12
z16
LVPGWTKPITIGR+XL_S
LVPGWTKPITIGR+XL_L
A_b_ion
A_y_ion
y6++
y9++
prec
b7+
y10+
y11+
m/z
IMNVIGEPIDERGPIKTK+XL_S
B_b_ion
B_y_ion
y11+
y13+
y8+
y12+
y14+
y3+
y10+
IMNVIGEPIDERGPIKTK+XL_L
y14++
y16++
b15+
y16+
